# Diagnosis of Pituitary Stalk Interruption Syndrome in a Newborn Presenting With Recurrent Hypoglycemia: A Rare Case Report

**DOI:** 10.7759/cureus.110888

**Published:** 2026-06-15

**Authors:** Fahad Albadr, Aljohara A Alwahhabi, Noura Alraeesi, Hussain Gamlo, Hayat Albadr, Hamad M Qabha

**Affiliations:** 1 Radiology, Medical Imaging and Neuroradiology, King Saud University Medical City, College of Medicine, King Saud University, Riyadh, SAU; 2 Internal Medicine, King Saud Medical City, Riyadh, SAU; 3 Radiology, Medical Imaging and Neuroradiology, King Abdulaziz Medical City, National Guard Hospital, Riyadh, SAU; 4 Radiology and Medical Imaging, Neuroradiology, King Fahd Military Medical Complex, Dhahran, SAU; 5 Radiology and Medical Imaging, King Saud University Medical City, College of Medicine, King Saud University, Riyadh, SAU; 6 Radiology, Medical Imaging and Neuroradiology, Dallah Hospital - Riyadh Joint Program, Riyadh, SAU

**Keywords:** congenital pituitary anomalies, ectopic posterior pituitary, hypopituitarism, neonatal hypoglycemia, pituitary stalk interruption syndrome (psis)

## Abstract

Pituitary stalk interruption syndrome (PSIS) is a rare congenital anomaly associated with multiple pituitary hormone deficiencies. It is characterized by an absent or thin pituitary stalk, an ectopic posterior pituitary, and a hypoplastic anterior pituitary and is typically diagnosed via MRI. The syndrome presents variably depending on age, with neonatal cases often manifesting as recurrent hypoglycemia. Early diagnosis is critical to prevent severe complications. We report a case of a preterm male neonate born via emergency cesarean section due to non-reassuring cardiotocography and vaginal bleeding in the setting of breech presentation. The neonate developed recurrent hypoglycemia despite glucose therapy. Endocrine evaluations indicated hypopituitarism, with low cortisol, growth hormone, and adrenocorticotropic hormone (ACTH) levels. MRI confirmed PSIS with a hypoplastic anterior pituitary, absent pituitary stalk, and ectopic posterior pituitary. Hormonal replacement therapy with hydrocortisone, levothyroxine, and growth hormone led to clinical stabilization. PSIS should be considered in neonates with unexplained hypoglycemia. Early MRI evaluation and prompt hormonal replacement therapy are vital for preventing complications and improving outcomes. Increasing awareness among clinicians can enhance diagnostic accuracy and optimize patient care.

## Introduction

Pituitary stalk interruption syndrome (PSIS) is a very rare congenital abnormality with an estimated occurrence of 0.5 per 100,000 births. It is characterized by a triad of anatomical abnormalities: a thin or absent pituitary stalk, an ectopically located posterior pituitary gland, and an absent or underdeveloped anterior pituitary gland [1]. PSIS is commonly detected on MRI, the primary diagnostic modality. First documented by Fujisawa et al. in 1987 following pituitary stalk transection surgery in ten patients with idiopathic pituitary dwarfism, they demonstrated an ectopic posterior pituitary on MRI [2]. The syndrome's precise pathophysiology is complex and not completely understood, as presentations can be varied and can occur at different stages of life. PSIS has also been associated with Breech presentation and difficult delivery, potentially due to pituitary stalk injury [3]. The clinical presentation related to endocrine hormone deficiencies can vary based on the age of onset [4]. Neonatal PSIS is challenging to diagnose, and delayed diagnosis can result in severe, life-threatening complications [4-5].

In our case, we encountered a newborn presenting with multiple episodes of hypoglycemia despite being treated with dextrose. The patient was then given hormonal therapy as the main line of management for the condition after diagnosis via MRI.

## Case presentation

This is a case of a preterm male neonate delivered by emergency cesarean section due to non-reassuring cardiotocography (CTG) and vaginal bleeding. The neonate was born in Breech presentation and was initially flat, apneic, and without a detectable heart rate. After one minute of resuscitation, the neonate began to cry weakly, regained a normal heart rate, and had labored breathing. The available perinatal history was otherwise limited, and detailed maternal history, APGAR scores, and complete birth anthropometric measurements were not available for retrospective review. He was eventually admitted to the neonatal intensive care unit (NICU) for prematurity, requiring respiratory support and treatment for hypoglycemia.

During his stay in the NICU, the neonate experienced three episodes of hypoglycemia. The first episode occurred on the first day of life with a blood glucose of 2.14 mmol/L and was managed with intravenous fluids (IV) D10%, resolving without further complications. However, with continuous monitoring and workup, another hypoglycemic episode occurred on day 22 of life, with blood glucose levels reaching 1.7 mmol/L, which was again managed with IV D10%. The following day, he experienced yet another episode of hypoglycemia, with his glucose level dropping to 0.37 mmol/L, requiring another dose of IV D10%.

A critical sample was obtained during the third episode of hypoglycemia, revealing the following results: glucose 0.37 mmol/L (N = 2.8-4.4 mmol/L), cortisol 16.5 nmol/L (N = 30-250 nmol/L), growth hormone (GH) 2.4 ng/mL (N = 5-40 ng/mL), adrenocorticotropic hormone (ACTH) 1.18 pmol/L (N = 10-60 pmol/L), C-peptide 0.26 nmol/L (N = 0.2-0.6 nmol/L), and insulin 1.14 mU/mL (N = 2.6-24.9 mU/mL).

On examination, the patient had normal male genitalia, bilateral descended testes, and no dysmorphic features were found. Further endocrine evaluations, including an ACTH stimulation test and assessment of the hypothalamic-pituitary axis (HPA), were performed as reported in Table 1. 

**Table 1:** Endocrine Workup Results Free T4: free thyroxine, TSH: thyroid-stimulating hormone, GH: growth hormone, FSH: follicle-stimulating hormone, LH: luteinizing hormone, ACTH: adrenocorticotropic hormone.

Table1: Endocrine Workup Results		Reference values
Free T4 (pmol/L)	7.10	8–25 pmol/L
TSH (uIU/ml)	2.58	0.8–12 µIU/mL
GH (ng/mL)	2.4	5-40 ng/mL
FSH (mIU/ml)	0.60	0.2–5 mIU/mL
LH (mIU/ml)	1.70	0.2–6 mIU/mL
Prolactin (mIU/L)*	1487*	500–6000 mIU/L
ACTH stimulation test		
ACTH (pmol/L)	0.52	10-60 pmol/L
Cortisol 0 min (nmol/L)	6.09	55–500 nmol/L
Cortisol at 30 min –> at 60 min (nmol/L)	76.6 – 66.30	Expected peak >360–500 nmol/L
* _Note: Prolactin is normal, which is somewhat atypical for PSIS (prolactin is often elevated due to loss of hypothalamic dopaminergic inhibition) _		

The ACTH stimulation test demonstrated low cortisol levels with a poor response, indicating central adrenal insufficiency. Thyroid function tests showed low T4 levels with inappropriately normal TSH levels, suggesting central hypothyroidism. Additionally, GH levels were low, indicating GH deficiency. Therefore, involvement of the adrenal, thyroid, and GH axes collectively strongly suggested hypopituitarism and made hyperinsulinism less likely

MRI of the pituitary gland and brain was performed using a dedicated pituitary protocol with thin-section sagittal and coronal T1-weighted imaging (Figure 1). This revealed a hypoplastic anterior pituitary gland and a 3-mm T1 hyperintense nodular focus at the median eminence along the proximal infundibular recess, representing the ectopic posterior pituitary gland. The pituitary stalk was not visualized. These overall findings were consistent with the diagnosis of pituitary stalk interruption syndrome. The optic nerves, optic chiasm, septum pellucidum, and corpus callosum were assessed and appeared normal, thereby excluding associated midline anomalies such as septo-optic dysplasia. However, the absence of representative imaging constitutes a limitation of this report.

**Figure 1:**
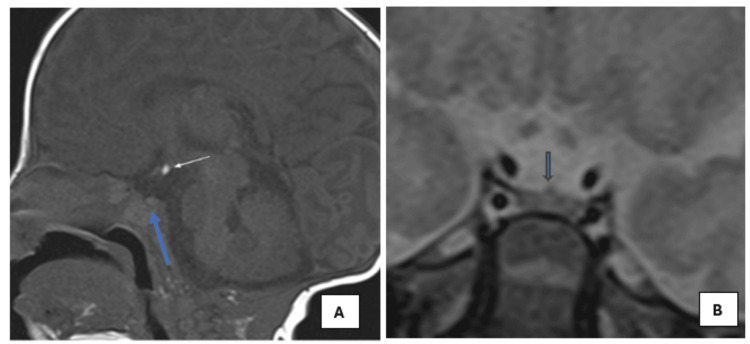
MRI of the Pituitary Gland (A) T1 sagittal MRI of the pituitary gland and brain, showing a hypoplastic anterior pituitary gland (blue arrow), and a 3-mm T1 hyperintense nodular focus at the median eminence along the proximal infundibular recess, which represents the ectopic posterior pituitary gland (white arrow). (B) T2 coronal MRI of the pituitary gland and brain, showing non-visualization of the pituitary stalk.

Immediate hormone replacement therapy was initiated following radiological confirmation of the diagnosis, consisting of hydrocortisone (3 mg/kg/day, IV divided every four hours), levothyroxine (25 mcg once daily), and GH injections (0.1 mg/kg every other day). Following the initiation of appropriate hormone replacement therapy, the patient remained stable, with no further hypoglycemic episodes recorded.

## Discussion

PSIS is an uncommon congenital disorder that may present during the neonatal period with recurrent hypoglycemia secondary to multiple pituitary hormone deficiencies [4,5]. In contrast, older children more commonly present with growth failure and short stature due to growth hormone deficiency [6]. Neonatal PSIS remains diagnostically challenging because of its rarity, nonspecific presentation, and poorly understood etiology, making MRI the cornerstone of diagnosis [7,8].

The classic radiological triad of PSIS consists of an absent or thin pituitary stalk, ectopic posterior pituitary, and hypoplastic or absent anterior pituitary gland [1,8]. Pituitary hypoplasia refers to incomplete development of the anterior pituitary gland, resulting in reduced gland volume on MRI and variable endocrine dysfunction involving the growth hormone, thyroid, and prolactin axes [9]. In contrast, pituitary aplasia describes complete absence of the gland and is more frequently associated with severe congenital endocrine deficiencies and developmental abnormalities [9].

MRI plays a central role in confirming the diagnosis of PSIS and excluding associated midline anomalies [1,8]. In our patient, MRI demonstrated a hypoplastic anterior pituitary gland, non-visualization of the pituitary stalk, and a T1 hyperintense ectopic posterior pituitary located at the median eminence, findings consistent with the classic PSIS triad. The intrinsic T1 hyperintensity of the ectopic posterior pituitary is believed to result from neurosecretory granules containing phospholipids and vasopressin-associated proteins [2]. Thin-section sagittal and coronal T1-weighted sequences are particularly sensitive for identifying the ectopic posterior pituitary bright spot and evaluating stalk integrity [2]. In addition, dedicated neuroradiological assessment should evaluate for associated midline abnormalities such as septo-optic dysplasia, agenesis of the septum pellucidum, corpus callosal abnormalities, and optic pathway hypoplasia [9]. In our case, the optic nerves, optic chiasm, septum pellucidum, and corpus callosum appeared normal.

The hypothalamic-pituitary-adrenal (HPA) axis plays an essential role in neonatal stress adaptation and glucose homeostasis [10]. Disruption of the HPA axis in PSIS contributes to recurrent hypoglycemia through cortisol deficiency and impaired counter-regulatory hormonal responses [10]. Similar endocrine abnormalities have been reported in neonatal and early infantile PSIS cases presenting with adrenal insufficiency, hypothyroidism, and growth hormone deficiency [11]. Furthermore, delayed diagnosis may result in persistent growth impairment and pubertal abnormalities later in life [12,13].

The etiology of PSIS remains incompletely understood, although both developmental and perinatal mechanisms have been proposed [4]. Several studies have suggested associations with perinatal complications, including breech presentation, birth asphyxia, and traumatic delivery [7]. In a study of 73 children with idiopathic growth hormone deficiency, breech presentation and perinatal asphyxia were identified as significant risk factors for hypopituitarism [7]. Genetic contributors have also been implicated, including mutations affecting pituitary embryogenesis such as LIM Homeobox 3 (LHX3) and other transcription factor genes [1].

Neonatal PSIS may present with diverse manifestations, including hypoglycemia, cholestasis, hypotonia, micropenis, cryptorchidism, and prolonged jaundice [3,14,15]. Atitallah et al. described neonatal PSIS presenting with hypoglycemia and cholestasis that improved following hormone replacement therapy [14]. Similarly, Boueilh et al. reported a neonate with hypotonia, micropenis, bilateral cryptorchidism, and severe hypoglycemia associated with congenital hypophyseal insufficiency and complete pituitary stalk interruption on MRI [15]. Unlike many previously reported neonatal cases, our patient presented predominantly with recurrent hypoglycemia without genital abnormalities or dysmorphic features, emphasizing that isolated metabolic manifestations may delay recognition of the underlying endocrine disorder.

The differential diagnosis of recurrent neonatal hypoglycemia includes congenital hyperinsulinism, adrenal insufficiency, inborn errors of metabolism, sepsis, and congenital hypopituitarism [16]. In our patient, suppressed insulin levels during profound hypoglycemia, together with low cortisol, ACTH, and growth hormone levels, made hyperinsulinism less likely and supported hypopituitarism as the underlying etiology.

Posterior pituitary hormonal dysfunction was not clinically evident in our patient. Serum sodium levels and urine output remained within normal limits throughout admission, with no clinical evidence suggestive of diabetes insipidus or syndrome of inappropriate antidiuretic hormone secretion (SIADH). This observation is consistent with previous reports demonstrating preserved posterior pituitary hormonal function despite ectopic posterior pituitary localization on MRI [4].

Early diagnosis and prompt hormone replacement therapy are essential for preventing recurrent hypoglycemia, adrenal crises, neurodevelopmental complications, and long-term morbidity [3,4]. In our patient, early endocrine evaluation combined with characteristic MRI findings enabled the timely initiation of hormone replacement therapy and stabilization of glucose levels. In addition, parental counseling regarding adrenal insufficiency and stress-dose hydrocortisone administration during intercurrent illness was provided before discharge as part of standard endocrine education.

A limitation of this report is that it represents a single case with limited longitudinal follow-up. Genetic testing was not performed, preventing assessment for developmental gene mutations associated with PSIS. Another limitation is the absence of representative MRI images demonstrating the described pituitary abnormalities and associated midline structures despite dedicated neuroradiological assessment. Furthermore, several baseline neonatal and maternal clinical parameters, including detailed maternal history, APGAR scores, and complete anthropometric birth measurements, were unavailable for retrospective review.

## Conclusions

PSIS should be considered in preterm neonates presenting with recurrent or persistent hypoglycemia, particularly when suppressed insulin levels and evidence of multiple pituitary hormone deficiencies accompany hypoglycemia. In our patient, the combination of low cortisol, ACTH, and growth hormone levels, together with characteristic MRI findings, enabled early diagnosis of PSIS before the development of more severe endocrine or neurodevelopmental complications. This case emphasizes the importance of recognizing a distinct biochemical pattern that should prompt dedicated pituitary MRI evaluation in neonates with unexplained hypoglycemia. Early endocrine assessment, timely neuroradiological diagnosis, and prompt initiation of hormone replacement therapy are critical for improving clinical outcomes. Although limited by the absence of genetic testing and long-term follow-up, this case contributes to the limited neonatal PSIS literature. It highlights the diagnostic value of MRI in identifying congenital pituitary abnormalities.
